# Cuspid Relationship and Pyorrhea Alveolaris*Read before the California Academy of Periodontology, San Francisco, California, October 18, 1922.

**Published:** 1923-03

**Authors:** Paul J. Boyens


### CUSPID RELATIONSHIP AND PYORRHEA ALVEOLARIS *

BY PAUL J. BOYENS, D. D. S.

The various writers on pyorrhea have advanced numerous theories as to the cause of this syndrome. While the contributions of most of these investigators are valuable to dental science, it seems very strange that so many should have failed to mention one very simple, yet vital, etiological factor of this disease, and that is the initial injury done to the peridental membrane by traumatic occlusion. Perhaps the reason that so many investigators have failed to lay much stress upon trauma as a vital factor, is because the disease can be satisfactorily accounted for through other causes, and furthermore, because trauma so frequently affects teeth that appear to be in perfect occlusion.

It is the purpose of this paper to review and classify briefly some of the less noticeable, but none the less severe types of traumatic relationship that can exist in full dentures which appear to be in perfect occlusion, and also to recall some of the other well-recognized factors.

It is admitted that lowered resistance and other expressions of constitutional weakness predisposes to this disease, but upon radiographic examination of any given case, we find that periclasia is almost always limited to certain teeth—sometimes it is the molars, in other cases it is the bicuspids, often it is the lower incisors or the upper laterals—with no uniformity of progress. The questions arise: "Why are not all teeth affected alike if it is a systemic disease, or why do we find pyorrheal lesions in patients that give every indication of having perfect health?" It is because the mechanical factor is more important

* Read before the California Academy of Periodontology, San Francisco, California, October 18, 1922.

than the systemic in causing extensive alveolar absorption. The unequal or abnormal stress of opposing teeth has been pointed out by Dr. Pague repeatedly at our monthly clinics during these past eight or ten years.

A study of the radiogram, the occlusion and occlusal wear of the affected teeth will bring out the significant relationship between the two, the teeth showing the most wear or abnormal wear—all other factors being equal—will be the ones that show the most extensive periclasia. A further study of the radiogram of the case in hand will almost always show some normal septa—septa that show none of the three cardinal pre-pyorrhea changes mentioned by Pollia, consisting of pericementitis—a thickening of the radiolucent line around the root—increased radiolucence of the alveolar septum, and a break in, or non-union of, the peridental lamellæ over the crest. It is the general rule that the cases which have the widest variations in the progress of the disease—that is, having both normal and hopeless teeth—are the ones in which the local or mechanical factors predominate, and conversely, those cases that show a uniform and moderate progress of periclasia of all the teeth, are the cases in which the systemic factors predominate.

The key to the harmonious relationship between opposing teeth lies in the normal function of the cuspids. They are designed by nature to act as "shock absorbers." "They guide and limit the movement of the mandible." They are strongly anchored and located at a comparatively great distance from the fulcrum. They are force deflectors and act as "guards" for the other teeth. However, it is not meant that they are entirely immune to periclasia, but they can withstand stress that would be traumatic to other teeth; because of their long root, pressure on them is resisted with less leverage.

How often do we see the lower incisors badly affected in a mouth that at first glance seems to have a normal occlusion, but let that patient go through the movements of the incising bite and ten chances to one it will be seen that the cuspids do not take any stress. Often the calcareous deposits—so common to this region—are blamed. While it is a factor, tartar alone rarely forms pockets—trauma causes the initial injury to the peridental membrane, and the ever-present bacteria are quick to make an assault on the weakened tissue. Trauma of this type is readily corrected by shortening the affected teeth to the merest contact. The original shape of the tooth should be restored as nearly as possible.

Again we will see a perfect set of teeth that shows extensive alveolar absorption around the palatal roots of the molars, where salivary deposits seldom form. To find the cause, note what happens during the balancing bite. The palatal cusps will invariably carry too much stress. Then notice the cuspids on the opposite side. More than likely they will be found to carry little or no stress. Their function is to prevent the other teeth from interlocking. Grinding the palatal cusps of the affected teeth should be done on the palatal slope, thus narrowing the tooth as well as shortening it. The final adjustment of the occlusion can be made automatically by the patients chewing "Svelto" paste of varying grits, which is so admirably adapted to this work; but it is well to remember that the loosened teeth move in chewing the grit, and are not ground as much as the rigid ones and therefore they need more disking. Entire contact between opposing teeth should never be lost—because the peridental tissue need the stimulus of function. The desired result is reached when the teeth glide smoothly over their opponents during the excursions of the mandible, and when the stress is redistributed to such a degree that the strongest teeth—especially the cuspids—carry most of the lateral stress.

Another very common type of trauma results in cases where all cusps interlock too closely. The powerful triturating movements produce too much lateral stress. The remedy for the locked bite by grinding—cuspids included

—and chewing grit paste, was given in detail by Dr. Ward at the meeting of the National Dental Association in Los Angeles.

The surrounding tissues of upper laterals are frequently traumatized during the incising bite by the strong and stubby lower cuspids. Grinding the laterals and the mesial angle of the lower cuspid will very readily correct this error.

Those dentures with the edge to edge bite would appear to be free from trauma, especially if all cusps are worn smooth, but they, too, suffer from trauma. In this type it will be seen that the cuspids do not limit the movements of the mandible. The patient will use "great force in mastication and make extreme movements" in trying to bring two sharp edges together in the shearing movements. A limited number of teeth will bear all the stress at one time, accompanied by great lateral and off-center stress. This type seems to be the most difficult to correct; it is accomplished, in a measure, by lessening the labio-lingual and bucco-lingual diameters of the occlusal surfaces by liberal grinding, by reproducing to some extent, the sulci and embrasures, and also by restoring any lost contact points. In this type it is especially advisable to have the patient avoid undue exertion during mastication. Many teeth are traumatized that do not appear to receive abnormal stress. During central occlusion or during the working bite it may not take place—but a close study of the extreme movements will usually show the abnormal pressure. Trauma is an all-important factor, yet certain stress that will cause trouble in one mouth may not in another. Certain patients seem to have a tendency or predisposition to pyorrhea. This predisposition is often due to a low resistance to this particular infection, for it must be borne in mind that periclasia is an infective process.

Of all the systemic factors, perhaps the tendency toward the soft salivary deposits is the most important. These deposits are composed, among other things, of a protein which has been claimed to be serum globulin.* This nitrogenous material makes a wonderful culture medium for parasites. Some patients' metabolism is such that their saliva is constantly saturated with this material, and, consequently, a film is being deposited during all periods of rest.

The much-maligned serumal deposits—the precipitated salts of the blood serum—are more often the result of inflamed gingivæ than the initial cause. This conclusion would seem logical, inasmuch as serum does not exude from healthy tissue and the initial inflammation must come from some of the other sources. Of course, neglect, improper brushing, rough enamel, short roots, over-strenuous chewing, frail process, etc., are also possible factors in this disease. These vary greatly in importance in different cases. But it may be well to summarize by stating that pyorrhea alveolaris is a chronic, auto-infective process, manifesting itself first radiographically in cervical pericementitis, and clinically in gingivitis; and terminating in degeneration and necrosis of the peridental membrane and absorption of the alveolar process—with or without visible pus. Its rate or progress depends upon various factors, the chief of which is abnormal stress of the opposing teeth, and this stress is exaggerated in those cases in which the cuspids fail to perform their function as shock absorbers and force deflectors.—*The Pacific Dental Gazette.*

